# Passive Removal of Silicone Oil with Temporal Head Position through Two 23-Gauge Cannulas

**DOI:** 10.1155/2016/4182693

**Published:** 2016-06-22

**Authors:** Zhong Lin, Zhi Sheng Ke, Qian Zheng, Zhen Quan Zhao, Zong Ming Song

**Affiliations:** The Eye Hospital, School of Ophthalmology and Optometry, Wenzhou Medical University, Wenzhou, Zhejiang 325027, China

## Abstract

*Purpose*. To report a new approach for removal of silicone oil.* Methods*. All surgeries were performed using 23-gauge vitrectomy system with two transconjunctival sutureless cannulas. At the beginning, most of the silicone oil was removed by traditional microinvasive vitrectomy system through inferior-temporal cannula. Then, the blood transfusion tube is removed from the inferior-temporal cannula, and the fluid-air exchange is performed. A passive fluid-air exchange was performed to aspirate the residual silicone oil after gradually turning the patient's head temporally by approximately 90° gradually.* Results*. After the surgery, all patients had a clear anterior chamber and vitreous cavity on slit lamp and B scan examination, respectively. The mean time taken for silicone oil removal and total surgery was 8.0 ± 1.4 minutes and 12.4 ± 2.5 minutes, respectively. The mean intraocular pressure 1 day, 3 days, 1 week, 1 month, and 3 months after surgery was 9.0 ± 5.8 mmHg, 11.3 ± 7.6 mmHg, 16.1 ± 6.9 mmHg, 17.7 ± 4.8 mmHg, and 17.1 ± 3.5 mmHg, respectively.* Conclusion*. This new approach may provide a safe and fast method to remove the silicone oil.

## 1. Introduction

The 23-gauge cannula transconjunctival sutureless vitrectomy has been widely used for treatment of vitreous and retinal diseases, including intraocular silicone oil removal. As compared to conventional 20-gauge cannula, the 23-gauge cannula system has some advantages, such as more secure surgical procedure and faster wound healing [1, 2]. New approaches for injecting and removing silicone oil in sutureless vitreoretinal surgery have been developed accordingly [1–5]. However, the silicone oil is difficult to be completely removed from the vitreous chamber even when using the 23-gauge cannula system. Many postoperative complications related to intraocular silicone oil have been reported, including secondary glaucoma [6], corneal endothelial decompensation [7], and intraconjunctival oil inclusion cysts [8]. Furthermore, the patients often complain about floaters, which interfere with their vision and quality of life. In order to remove the silicone oil and emulsified silicone oil drops completely, we report a new surgical approach with temporal head positioning and passive fluid-air exchange through two 23-gauge cannulas.

## 2. Methods

Twenty-four silicone oil eyes of 24 patients, from the Eye Hospital of Wenzhou Medical University, were enrolled between December 2013 and June 2014. The study followed the tenets of the Declaration of Helsinki and was approved by the Ethics Committee of Wenzhou Medical University. All participants signed a written and informed consent.

All patients with primary vitreous or retinal disease, such as vitreous hemorrhage, rhegmatogenous retinal detachment, and proliferative vitreoretinopathy, who previously underwent 23-gauge transconjunctival sutureless vitrectomy and silicone oil (5,000 cSt) injection in the Eye Hospital of Wenzhou Medical University, were included in this study. Silicone oil was used as tamponade for all eyes for at least 3 months before removal. The binocular indirect ophthalmoscope, optical coherence tomography (OCT), and B-scan all indicated that the retina was attached preoperatively.

**Figure 1:**
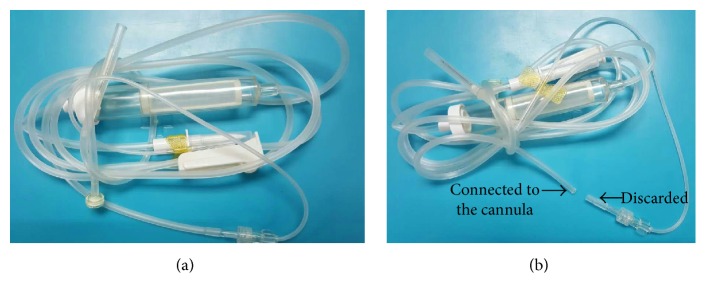
(a) Blood transfusion tube (model: IS-V9; Shanghai Kindly Enterprise Development Group, Shanghai, China). (b) The adaptor of the blood transfusion tube was cut. One side of the blood transfusion tube was connected to the inferior-temporal cannula after removing the valve.

**Figure 2:**
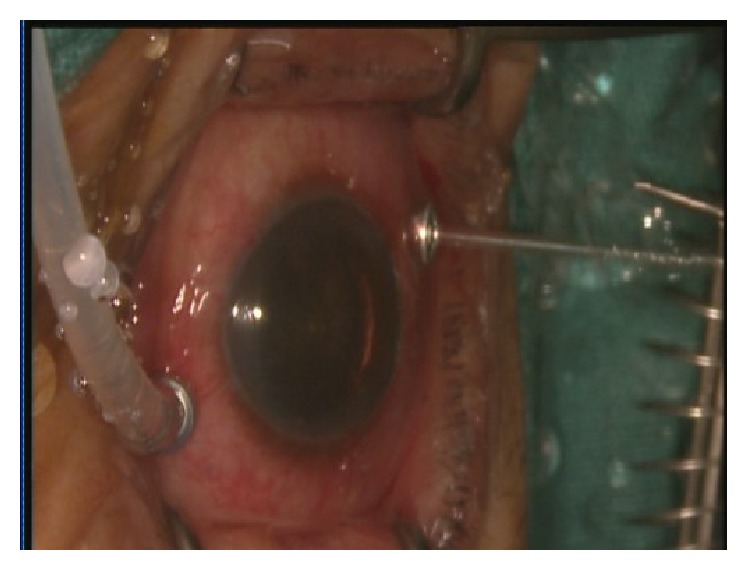
The passive fluid-air exchange was performed after removing the blood transfusion tube from the inferior-temporal cannula. At the same time, the patient's head was gradually turned temporally by approximately 90°.

All procedures were performed by the same surgeon (Zhi Sheng Ke) using 23-gauge (Midlaps, US) valve casing transconjunctival sutureless vitrectomy system after retrobulbar anesthesia with a 50% mixture of 2% lidocaine and 0.75% bupivacaine. To begin the surgery, two 23-gauge cannulas, that is, the superior-nasal infusion cannula and the inferior-temporal operation cannula, were established. A trocar was inserted into the intended sclerotomy site at an angle of approximately 30° parallel to the limbus with the bevel up. Once past the trocar sleeve, the angle was made perpendicular to surface and the cannula was inserted into the eye. A scleral tunnel incision was made. The cannula was held in place with forceps, and then the trocar was removed. The infusion tube was attached to the superior-nasal cannula. The infusion was opened and adjusted to approximately 60 cm. The adaptor of a blood transfusion tube (model: IS-V9; Shanghai Kindly Enterprise Development Group, Shanghai, China) was cut with scissors (Figure 1). The inferior-temporal cannula valve was removed and connected to one side of the blood transfusion tube. The diameter of the cannula without the valve was 1.96 mm, and the diameter of the blood transfusion tube was 2.00 mm. The other side of the blood transfusion tube was connected to the vitrectomy instrument. The negative pressure of the vitrectomy instrument was adjusted to approximately 600 mmHg (1 mmHg = 0.133 kPa). The silicone oil removal was started through the blood transfusion tube. During the oil removal, anterior chamber wash was performed to assist the removal of oil drops when necessary. After that, the fundus status was examined by inserting a 23G light probe. Additional procedures, such as endolaser and membrane peeling, were performed as needed through the cannula(s). If the fundus was in good condition, the blood transfusion tube was removed from the inferior-temporal cannula. At the same time, the patient's head was gradually turned temporally by approximately 90°. A passive fluid-air exchange was started to remove the residual silicone oil as well as balanced salt solution (BSS) out of vitreous cavity (Figure 2). The air irrigation pressure was reduced to 10–15 mmHg. At this time, the temporal sclera and cannula were observed directly, since they were turned out of the visual field of microscope. Finally the superior-nasal cannula was removed. The scleral incisions were pressed with cotton swab to close them. If leakage was noticed and continued beyond 1 minute, a suture was placed to close the wound.

The time taken for silicone oil removal and total surgery was recorded. Postoperative residual silicone oil in the anterior chamber and vitreous chamber was verified by slit lamp examination and B-scan, respectively, within 2 days after surgery. Preoperative IOP and postoperative IOP at one day, three days, one week, one month, and three months were recorded. Patients' postoperative complaints were also recorded.

All statistical analyses were performed with Statistical Analysis System for Windows version 9.1.3 (SAS Inc., Cary, NC). A *P* value of <0.05 was considered statistically significant.

## 3. Results

Overall, 10 male and 14 female patients were included. The average age was 50.5 ± 18.2 years. The mean time interval between pars plana vitrectomy with silicone oil tamponade and removal of silicone oil was 3.5 ± 0.8 months. Fifteen eyes were aphakic, 8 eyes were pseudophakic with an intact posterior capsule, and 1 eye was phakic.

**Figure 3:**
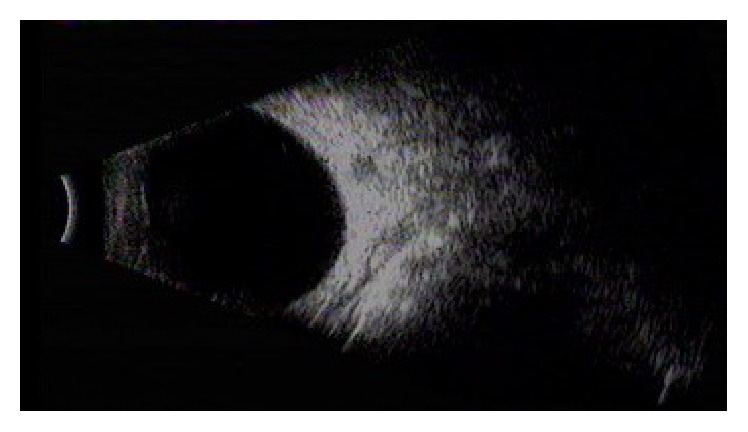
B-scan ultrasonography showed a clear vitreous cavity of a patient after the surgery of silicone oil removal.

Silicone oil was removed successfully in all these 24 cases. No severe postoperative complications were noted, such as severe decrease of intraocular pressure, corneal edema and opacity, intraocular tissue damage, intraocular hemorrhage, and retinal detachment. Postoperatively, all the patients had a clear anterior chamber and vitreous cavity on slit lamp and B-scan examination, respectively (Figure 3). One patient with sutured scleral incisions complained of foreign body sensation (4.2%) and 1 (4.2%) patient complained of floaters postoperatively.

The mean time taken for silicone oil removal and total surgery was 8.0 ± 1.4 minutes and 12.4 ± 2.5 minutes, respectively. Besides, 8 eyes underwent posterior capsulotomy, intraocular lens was implanted in 10 aphakic eyes, 1 eye underwent phacoemulsification, 7 eyes underwent an additional cannula to peel epiretinal membrane, and 6 eyes underwent retinal laser photocoagulation.

The mean IOP before surgery and 1 day, 3 days, 1 week, 1 month, and 3 months after surgery was 13.7 ± 3.9 mmHg, 9.0 ± 5.8 mmHg, 11.3 ± 7.6 mmHg, 16.1 ± 6.9 mmHg, 17.7 ± 4.8 mmHg, and 17.1 ± 3.5 mmHg, respectively. At 3 months postoperatively, the BCVA improved compared to that preoperatively (Snellen, 0.28 ± 0.31 versus 0.12 ± 0.15).

## 4. Discussion

Previous studies reported that residual silicone oil may cause keratopathy [7] and secondary glaucoma [6] and even migrate along the intracranial portion of the optic nerve and into the lateral ventricles of the brain [9]. Therefore, complete removal of silicone oil is important. Compared to conventional 20-gauge cannula transconjunctival suture to remove silicone oil, silicone oil removal by 23-gauge cannula transconjunctival sutureless vitrectomy system has some advantages, such as better eye closure and less incision leakage [1, 2]. However, the main problem of silicone oil removal is still the residue of silicone oil.

**Figure 4:**
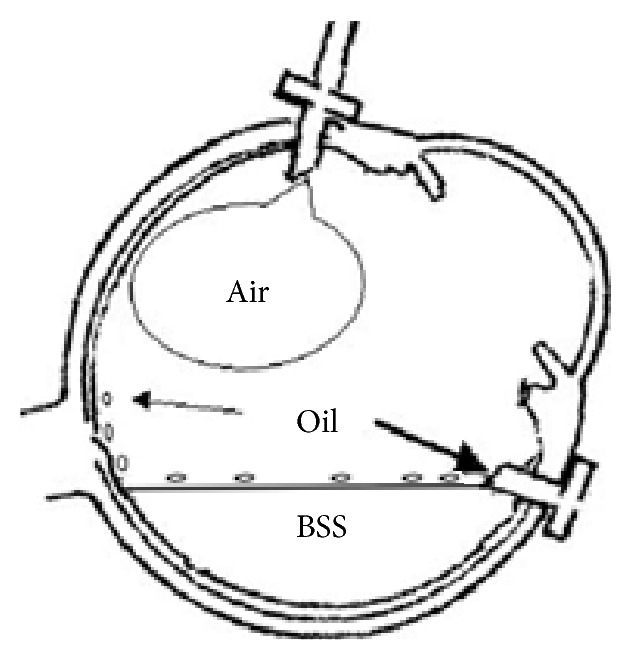
The emulsified silicone oil droplets adhering to the retinal surface would be pushed into the vitreous cavity, while the air entered into the vitreous cavity. The silicone oil droplets would float up because of buoyancy and finally form a thin oil layer between the air (upper layer) and the balanced salt solution (BSS, bottom layer). At that time, the patient's head was gradually turned temporally by approximately 90°, so that the silicone oil layer together with BSS would flow out of the eyeball through the inferior-temporal cannula due to gravity.

In this study, we proposed an improved method for silicone oil removal. Most of the silicone oil was removed by traditional microinvasive vitrectomy system. The principle behind this method is related to the physical property of silicone oil itself. First, the silicone oil easily floats above water, since its density (0.97 g/cm^3^) is lower than water (1.0 g/cm^3^) [10]. Second, the silicone oil tends to form oil droplets above water when it coexists with air, since its surface tension is lesser (35 mN/m) than that of air (approximately 80 mN/m) [10]. Third, silicone oil droplets tend to fuse together above water, since it has high viscosity (5000 cSt) [10]. The present silicone oil removal approach was performed not only through 23G transconjunctival sutureless vitrectomy system, but also combined with the passive fluid-air exchange. At the beginning of the passive fluid-air exchange operation, the vitreous cavity was filled with BSS. The residual silicone oil drops at the bottom of the vitreous cavity rose up, while the BSS went into the vitreous cavity. The emulsified silicone oil droplets adhering to the retinal surface would be pushed into the vitreous cavity, while the air entered into the vitreous cavity. The silicone oil droplets would float up because of buoyancy and finally form a thin oil layer between the air (upper layer) and the BSS (bottom layer). At that time, the patient's head was gradually turned temporally by approximately 90°, so that the silicone oil layer together with BSS would flow out of the eyeball through the inferior-temporal cannula due to gravity (Figures 2 and 4).

In this study, the mean time intervals of silicone oil removal and total surgery were 8.0 ± 1.4 minutes and 12.4 ± 2.5 minutes, respectively. Since the passive fluid-air exchange of this approach does not require the use of corneal contact lens, noncontact observation lens, vitrectomy probe, or backflush needle, the time is apparently shorter than traditional method. After surgery, all patients had a clear anterior chamber and vitreous cavity. Besides, only 1 (4.2%) patient complained of floaters postoperatively. Moreover, no severe intra- or postoperative complications were noted. Postoperative intraocular pressure remained stable, and the BCVA improved. However, the small sample size, lack of controlled group, and relatively short follow-up time are the limitations. Large controlled samples and long-period follow-up are warranted.

There were several advantages of this new approach besides less residual of silicone oil. First, the intraocular pressure was stable intraoperatively. Second, there is no need for the vitrectomy probe or backflush needle to be inserted into the vitreous cavity during the fluid-air exchange operation of this approach. Hence, it reduces the change of lens, choroid, and retinal injury. Third, the fluid-air exchange operation can be performed even without transparent refractive media. This is especially advantageous in patients with corneal lesion.

In summary, this new approach of silicone oil removal uses negative pressure of traditional microinvasive vitrectomy system to aspirate most of the silicone oil. The technique also uses passive fluid-air exchange to remove the remaining emulsified silicone oil droplets. This new approach is safe, facilitated, fast, and with wider indications.
